# The Emperor of Fashion’s New Starts: Creativity and Meaning in Life in Karl Lagerfeld

**DOI:** 10.5964/ejop.4521

**Published:** 2021-08-31

**Authors:** Claude-Hélène Mayer, James L. Kelley

**Affiliations:** 1 Department of Industrial Psychology and People Management, University of Johannesburg, Johannesburg, South Africa; 2 Faculty of Cultural Studies, European University Viadrina Frankfurt (Oder), Frankfurt, Germany; 3 Independent Researcher, Norman, OK, USA; University of the Free State, Bloemfontein, South Africa; Nelson Mandela University, Port Elizabeth, South Africa

**Keywords:** creativity, meaning in life, Four C-model, Big-C, Pro-C, little-c, mini-c, WICS, psychobiography, fashion world, haute couture

## Abstract

During his lifetime, Karl Otto Lagerfeld (1933–2018) attained such industry renown that he became widely known as the Emperor of Fashion. Lagerfeld ran several fashion houses, such as Chanel and Fendi, leading them to unprecedented profits. He also created his own fashion label. Owing to his unremitting pursuit of excellence through creative expression, Lagerfeld’s creativity, energy and intuition for fashion trends seemed only to expand throughout his long career. The authors suggest that, through his creative approach to fashion, architecture, and publishing, Lagerfeld articulated and refined a core set of values-such as “Bildung,” “lightness” and “the unexpected”—that served as a Diltheyan “nexus” linking the Prussian-born designer with the global consumer. The authors apply two specific creativity theories to Lagerfeld’s life and work, namely the mini-c, little-c, Pro-c and Big-C creativity theory and Sternberg’s WICS-model (wisdom, intelligence and creativity). The article uses a psychobiographical case study design formulated according to a research paradigm of modern hermeneutics. First- and third-person data on Lagerfeld were collected and evaluated through a hermeneutically-informed syntho-analysis. Research ethics were followed. The findings demonstrate the interplay of mini-c, little-c, Pro-c and Big-C creativity throughout the subject’s lifetime, as well as the subject’s application of WICS, both of which led to the subject’s worldwide success. Conclusions are drawn and recommendations for future research and practice are provided.

*What I like best in life is new starts.*

— *Karl Lagerfeld*

Creativity emerged as a popular research topic in the middle of the last century (Guilford, 1950), and has since become a prominent theme in a wide array of fields such as psychology and sociology (Amabile, 2018; Damian & Simonton, 2015; Runco & Albert, 2010). Ever since its rise to prominence in academe, creativity has also been closely linked with psychobiography (Johnson, 1985; John-Steiner, 2015; Kováry, 2011, 2016; Latilla & Kramer, 2018; Mayer, 2017; Mayer & van Nierkerk, 2020). During the past decades, psychobiographers have explored creativity at work in extraordinary, imaginative individuals, such as lyricists, writers, psychologists, and politicians (Schultz, 2005). In particular, these researchers have focused on the characteristics, development, and expression of creative individuals across the life span (Holm-Hadulla, 2012; Kasser, 2013; Kováry, 2011; Mayer, 2017; Mayer & van Niekerk, 2020; McAdams, 1988; McRunyan, 2005; Osorio, 2016; Ponterrotto, 2015; Runco & Albert, 2010). Creativity has not only been considered in its positive aspects, but also in its challenging facets, such as mental disorder and psychopathology (Belli, 2009; Holm-Hadulla, 2018).

Creativity is the skill to develop original ideas that are generally valuable and useful (Sternberg & Lubart, 1995). The creative individual’s work is characterised by its originality, but also by its effectiveness (Runco & Jaeger, 2012). Creativity develops across the life span (Hilton, 2008; Trilling & Fadel, 2009), helping individuals to cope with life challenges at every phase of personal development (Romero, Hyvönen, & Barberá, 2012). Many factors work together to determine the shape creativity will take, such as the genetic predispositions of the creator, as well as the type of environment in which the creator finds herself, and also the context that informs each creative endeavour (Csíkszentmihályi, 2014). These factors are operative from the earliest stages of the development of the creative skill, to their full flowering. In Lagerfeld’s case, throughout his childhood and early adult years he constantly oscillated between viewing artworks and fashion magazines, and translating what he saw into sketches of outfits. This basic process continued throughout his entire life, though it was doubtless refined as time wore on. Creativity research supports what Lagerfeld’s specific experience bore out: Not only at its root, but also throughout its development, creativity seems to be strongly connected to meaning-making and meaning in life (Kaufman, 2018a, 2018b).

Meaning in life is a concept that encompasses not only life’s inherent value (Adler, 1964), and the framework through which life can be interpreted (Frankl, 1963), but also the sense of achievement in meeting goals (Seligman, 1991) and an enthusiastic orientation that views life as exciting, interesting, or engaging (Rogers, 1951). Reker (2000) defines the meaning of life as a “multidimensional construct consisting of the cognisance of order, coherence, and purpose in one’s existence, the pursuit and attainment of worthwhile goals, and the accompanying sense of fulfillment.”

The present writing explores creativity and meaning in life as they manifested in Karl Lagerfeld, the German-born, Paris-based creative fashion designer, photographer and artist who lived and worked for most of his life in France (Langkjaer, 2019). Lagerfeld, born in 1933, achieved iconic status as the “emperor of fashion” in the 20th and 21st century by building up several fashion houses, such as Chanel, Fendi, and his own eponymous brand, all of which he led to unprecedented success (Hall, 2019). Lagerfeld passed on in 2018; he has, however, left a legacy as a highly creative, innovative and extravagant designer and artist (Hall, 2019).

Owing to his unremitting pursuit of meaning in life through creative expression, Lagerfeld’s intuition for fashion trends seemed only to expand throughout his long career. Matheson (2016) points out that creativity impacts on the meaning of life, and vice versa, thereby increasing mental health and well-being. This article suggests that Lagerfeld generated meaning in his life through his creative approach to fashion, architecture, and publishing.

After having discussed the interlinkages of creativity and meaning in life, the authors apply two creativity theories to Lagerfeld’s life and work, namely Beghetto, Glăveanu and Runco’s mini-c, little-c, Pro-c and Big-C creativity and Robert Sternberg’s WICS-model (wisdom, intelligence and creativity).

## Creativity and Meaning in Life

Creativity bolsters the capacity to cope with challenging situations (Romero et al., 2012), and also contributes positively to mental health and well-being (Forgeard, 2013; Simonton, 2014). According to Csíkszentmihályi (1997), creativity affords positive development by transforming an existing sphere of activity into a new, more inclusive shape. In Lagerfeld’s case, his creativity transformed the fashion and design industry from a merely reactive and nostalgic domain into one that established forward-looking trends and concepts (Bacqué, 2020; Hall, 2019).

Creativity is not a free-floating attribute that can be segregated either from the process through which it is enacted or from the products that are its result; even less can we consider creativity apart from the lives of those who bring it into being (Sak & Oz, 2010; Sternberg, 2006). Indeed, at creativity’s core is an interplay between 1) the personality of the creative person who juxtaposes divergent perspectives into a unified, bounded product (Csíkszentmihályi, 2007; Glăveanu, 2015), and 2) the socio-cultural milieu in which the creative situation is framed (Amabile, 1983; Glăveanu, 2015; Glăveanu & Sierra, 2015; Helfand, Kaufman, & Beghetto, 2016; Kaufman & Beghetto, 2013).

Kaufman (2018b) points out that creativity can help individuals to lead happier, more fulfilling lives by linking their own efforts to lead the good life to global issues of perennial concern (Kaufman, 2018b). For Sääksjärvi and Gonçalves (2018), creativity is built upon three essential components: meaning, novelty and utility. Bock (2016) supports the hypothesis that basic creative activity, such as drawing and journaling, not only fosters meaning-making in an individual’s life, but also can serve as a basis for generating socially relevant content. If individuals have the capacity to express themselves freely and can chose their mode of expression, agency and voice can be established through creative meaning-making (Bock, 2016).

## Creative and Meaningful Leadership

Over the course of many decades, Karl Lagerfeld influenced and even led the *haute couture* and fashion industry to new heights of creative expression. Sternberg (2005) has emphasised that extraordinary and effective leadership synthesizes three qualities: wisdom, intelligence and creativity (the WICS model). Wisdom is the ability 1) to use intelligence, creativity and knowledge in a manner that reflects a greater common good; 2) to balance intra-, inter- and extra-personal interests; 3) to adopt a long-term perspective and include well-defined value sets; 4) to recognise problems of injustice; as well as 5) to apply dialogical thinking that considers problems from multiple perspectives. Intelligence refers to the contextualized ability to plan, monitor and evaluate problem solutions, define performance components and execute solutions while acquiring knowledge and the ability to solve problems through the allocation of resources. Finally, creativity is the skill to generate novel, high-quality ideas and products.

Sternberg (2005) further differentiated between skills and types of creative leadership. The nine skills underlying successful leadership in a field are: redefining and analyzing problems, selling solutions, taking sensible risks, surmounting obstacles, believing in one’s ability to accomplish the task at hand, tolerating ambiguity, finding extrinsic rewards for the things one is intrinsically motivated to do, and avoiding stagnation by continuing to grow intellectually.

The eight types of creative leadership (Sternberg, 2005) are: replication (maintaining the field or organization where it is), redefinition (viewing the status of the organization from a different point of view), forward incrementation (leading a field or organization in the direction it is already going), advance toward incrementation (moving a field or organization in the direction it is already going, but beyond the expected rate of progression), redirection (redirecting an organization or field toward a different direction), reconstruction (moving a field or organization back to where it once was), reinitiation (moving a field or organization from a new starting point in a direction different from the one previously pursued), and synthesis (integration of ideas that were previously seen as unrelated or opposed).

The viability of creative individuals’ output is reflected in whether or not the narratives they create inspire others to succeed (Sternberg, 2005, 2008). It follows that successful leadership narratives enable innovators to fulfill their mission by realizing their creative potential. These success-breeding stories both align with the extraordinary individual’s action and relate to the life events and emotions of their followers. Stories that are creative, but fail to match the follower’s needs are not persuasive, and thus fail disseminate the leader’s values (Press, McLean, & McCauley, 2020; Sternberg, 2005).

## Big-C, Little-c, Pro-c, and Mini-c Creativity in Creating a Meaningful Life

Ronald A. Beghetto’s and James C. Kaufman’s Big-C creativity theory highlights extraordinary individuals who create extraordinary products (Beghetto, 2010; Beghetto & Kaufman, 2007, 2009; Glăveanu & Sierra, 2015; Runco, 1995). Later, theorists appended three other components to Big-C, which we will outline below: little-c, Pro-c, and mini-c (Beghetto & Kaufman, 2007, 2009).

The products of Big-C creativity are exceptional, long lasting, and socially relevant (Glăveanu & Sierra, 2015); they are exemplified by the work of Picasso, Mozart, Dickinson and Einstein (Beghetto, 2010). This category of creativity is often favoured in Western creativity research (Glăveanu & Sierra, 2015), since it presents a tangible product (Beghetto, 2010; Runco, 2007) that is recognised, valued (Gralewski & Karwowski, 2016; Klausen, 2010), and verified by the audience (Kasof, 1995).

In contrast with Big-C creativity (genius creativity) stands little-c creativity (everyday creativity), typified by small-scale, basic products originating at school, the home, or the workplace (Kaufman & Beghetto, 2009, p. 3). Between these two categories, Kaufman and Beghetto (2009) placed Pro-c (expert creativity) to indicate a lower-impact, smaller-scale level of creativity that, despite falling short of Big-C’s genius, nonetheless exhibits marked facility and worthwhile innovation. Most professionals with a creative flair are little-c creators, and only a handful will ever qualify as Pro-c creators. A fourth level of creativity was also proposed by Beghetto and Kaufman-mini-c creativity-which, like Pro-c, is sandwiched between Big-C and little-c. Mini-c is subjective, being comprised of daily creative processes, which, according to Kaufman and Beghetto (2009) include “the novel and personally meaningful interpretation of experiences, actions, and events” (Kaufman & Beghetto, 2013). Researchers agree that to develop Big-C creativity, preparatory acts of Pro-C and mini-c creativity are required (Kaufman & Beghetto, 2009, 2013). In support of this developmental line of thinking, Harrison (2016) has presented an applied analytical autoethnography of a songwriter and his journey from little-c to Pro-c creativity. Rowles (2017) has helped to further differentiate Beghetto’s and Kaufman’s componential theory by providing evidence that creativity is perceived differently across the four C’s: while mini-c’s meaning centers on the individual, Big-C’s meaning often fans out to effect social groups and even the entire globe (Rowles, 2017).

## The Aim of the Study

The aim of the present study is to construct a unique perspective on Karl Lagerfeld by exploring how his creativity guided his life and work. The study follows Elms’ (1994) method of holistic life reconstruction, but does so with the full knowledge that any such reconstitution can only be partial, since the authors chose a specific psychobiographical theory and highlighted selected core events in his life. It is assumed that Lagerfeld’s creative activity was instrumental in his achievement of a meaningful life. The article therefore fits into this special issue on psychobiography as an exploration of creativity as a life-meaning generator.

## Method

Psychobiography was chosen as a guide to the authors’ exploration of Karl Lagerfeld’s life and work (Jareño Gómez, Chiclana Actis, & Noriega García, 2019). The study focuses on a single case and explores the life, creativity and creative work of an extraordinary individual across the life span (Gruber & Bödeker, 2005), following Long’s (2014) call for more substantive qualitative studies in creativity research.

### Research Method and Paradigm

In recent years, psychobiography has become more commonly utilized as a research method (Kováry, 2011) to explore the lives of extraordinary individuals (Alexander, 1990; Fouché & Van Niekerk, 2005, 2010) by applying specific psychological theories to reconstruct their lives from new and original perspectives. Theories on creativity have been applied previously in psychobiographical research (John-Steiner, 2015; Kováry, 2011; Mayer, 2017; Mayer & van Niekerk, 2020; McRunyan, 1988, 2013) and are explored here further with regard to meaning in life. In terms of the theoretical frame, this study fits within the context of positive psychology. It examines positivity in the life of Lagerfeld through the theoretical lenses of creativity (Seligman, 2019) and meaning-making (Wong, 2011, 2019). This study thus contributes to the positive psychology approach recently promoted as part of psychobiographical approaches (Mayer, 2017). Along with positive psychology and the creativity theories used in this study, hermeneutics acknowledges that values are constructed by individuals who encode their socially-influenced meanings into gestures, attitudes, speeches, writings and other signs that can only be decoded by investigators who bring their own experiences to bear on the process of interpretation. Thus, the present writing, as a hermeneutically-attuned psychobiography, follows Dilthey in assuming that positivity, creativity and intelligence are not objectively definable in same way that the natural sciences measure regularities in the material world (Dilthey, 1956, p. 3).

Following Dilthey's (1997, 2002) modern hermeneutics, the authors take up a holistic approach to understand creativity in the context of personal development across a lifespan, holding together a dual focus on life processes and life events. Lagerfeld’s creative activity is examined according to Dilthey’s hermeneutically-aware “Verstehen” approach, which provides a more holistic picture by yielding both emic and etic insights (Dilthey, 2002; Kováry, 2011, 2015; Nortjé, Fouché, & Gogo, 2013). Our Diltheyan approach to the materials examined about Lagerfeld-which pays heed to the deeper motivations submerged both in the texts themselves as well as in *us*, the authors, as outsiders to the meanings embedded therein-allowed us to avoid a simplistic, flat reading of the data. For example, we cannot take at face value Lagerfeld’s oft-quoted remark about his creative ideas arising spontaneously while he relaxes in the bathtub: “I saw it in a dream, [and] put it on my paper” (Karl Lagerfeld transforms Grand-Palais, n.d.). Instead, we must take into account the subject’s own construction of meaning, what meaning means to him, all the while understanding that we, the investigators, are always at the mercy of our own submerged presuppositions about meaning creation. Lagerfeld was steeped in the Neo-Romantic aesthetics of Kandinsky and Baudelaire (WSJ Magazine, 2020), and thus appealed to pure inspiration to distance himself from his influences. However, Lagerfeld used this notion of Romantic productivity (Schmitt, 1998) to turn consumers’ attention to the emotional effect his creations evoke, rather than getting bogged down in pedantic, heady questions about art history (Givhan, 2019).

### Karl Lagerfeld in Perspective

Karl Lagerfeld was chosen purposefully as the subject of research (Elms, 1994) on account of his outstanding creativity, which he displayed through his paradigm-shifting work as a fashion designer (Langkjaer, 2019), an example being his unique collaboration with the Swedish mass-market retailer H&M (Rollet, Hoffmann, Coste-Manière, & Panchout, 2012; Rosa, 2014). Lagerfeld’s influence stretches far beyond the world of fashion design, however, leaving its mark in politics, aesthetics, and entrepreneurialism (Bacqué, 2020) as when he staged a “runway protest” in 2014, which featured models holding picket signs sporting slogans such as “History Is Her Story” and “Be Your Own Stylist” (Givhan, 2019). This spectacle provoked fresh discussion in the media about gender and the political implications of the fashion industry. As of the time of this writing, no psychobiography on Karl Lagerfeld was found.

### Data Collection, Analysis, and Reporting

Data was collected through first-person and third-person documents (Allport, 1961). The former included autobiographical accounts, internet sources, journal and newspaper articles, public interviews, video documentaries and selected Lagerfeld products (AlpinRunner, 2021). Among the third-person documents were biographies, interview accounts and case studies (Ellington, 2018; Langkjaer, 2019; NYT, 2015).

The authors followed the content analysis of Krippendorff (2018), including the collection of information with respect to the subject (step 1), creation of topics (step 2), coding of information (3), categorization and labelling of the content (4), and (5) creation of dissected meaning characteristics. Our content analysis was aided by our overarching hermeneutical approach, as when we linked Sternberg’s three characteristics-1) Creativity, 2) Intelligence, and 3) Wisdom-into a hierarchy that encompassed, respectively, 1) everyday creativity, 2) interpersonal relations, and 3) ideological or value-oriented sphere. Such a structuring follows Dilthey’s and other hermeneutics scholars’ insistence on integrating an understanding of the subject’s own socio-cultural constructions of meaning into the analysis of said subject’s life. The presentation of the findings was guided by the qualitative analysis process (Yin, 2013).

### Ethical Considerations

Customary ethical standards for psychobiographical research were followed by the authors, leading them to explore the subject of research in an ethical, respectful, empathetic and accountable manner (Elms, 1994; Ponterrotto, 2015; Schultz, 2005).

## Findings and Discussion

This section will examine the WICS model of leadership and the Big-C, Pro-c, little-c, and mini-c theory as it played out in the life and work of Karl Lagerfeld.

### WICS Model of Leadership and the Big-C, Pro-c, Little-c, and Mini-c Theory

Each of the components of Sternberg’s WICS leadership model can be found to be at work at different junctures in Karl Lagerfeld’s life. Creativity is displayed in Lagerfeld’s constant generation of new ideas; intelligence is evinced in his ability to arrange these new ideas into constellations that form the basis for products that the public finds compelling; wisdom comes into play for Lagerfeld at those junctures when he has taken care to craft his productions with the values of the wider public in mind. In the present essay, the synthesis of these three components (WICS) have been conceived as an ideal type of the creative process, progressing from the mini-c and little-c beginnings-the everyday creative steps that generate the raw materials for creation-to the Pro-c and Big-C levels. In the latter, works and projects are crafted and brought to fruition through the institutions and channels necessary to bring them into being.

### Creativity

At the level of creativity, raw ideas are called forth from everyday experience and are provisionally framed for use in later, higher-level refinements. If an individual does not possess this spontaneous fount of creativity, she has nothing to process, no bricks and no mortar with which to build. Young Karl was brought up in the rural area close to Hamburg, Germany, where he busied himself reading books and drawing-creative activities that he had chosen early in his life which fostered his imagination and creativity-far removed from social contact of his siblings and other children (AlpinRunner, 2021). It was not only in his youth, however, but also throughout his life, that “[Lagerfeld felt] an inner compulsion to find out everything there is to know, read everything; the curiosity [was] ceaseless” (Colapinto, 2007, pp. 114–115 cited in Langkjaer, 2019, p. 4). It is here, in his adult years, that we can find evidence of the WICS creative sphere as posing a specific challenge. Lagerfeld seems to have been tempted to allow his creative work to swallow his private life, turning even close friendships into grist for artistic productivity. Though he often broke ties with associates such as Anna Piaggi once they outlived their usefulness, he nonetheless showed concerns for his companion Jacques de Bascher as well as for his seamstresses that went far beyond utility (Bacqué, 2020). Overall, Lagerfeld’s tendency to overwork was counterbalanced by his constant attempt to turn his life into a vessel overflowing with conceptions that could be turned into items that were marketable and thus shared by a broad swathe of the public.

### Intelligence

Lagerfeld’s intelligence is evinced through his ability to frame his creative ideas so that they follow a well-worn aestheticist path by bringing seemingly distant elements together in surprising juxtaposition. Without a deep knowledge of every phase of modern culture-from Frederick the Great’s era to that of Dalì-Lagerfeld would have had no basis for making his bold fashion moves. One of the German designer’s apt insights was that people want to buy products that pull them away from the workaday, “bourgeoise” sphere, though without placing them squarely in either the dressed-down, ragamuffin, lower-class category, or its opposite, the icy, aloof, humorless category of the rich. This tightrope walk was performed by Lagerfeld in many variations, but a prominent one is his use of the theme of “lightness.” As one commentator put it: “Lagerfeld’s particular genius was in bringing a feelgood factor to the traditionally chilly world of Parisian elegance” (Cartner-Morley, 2019). The opulent aura of Joan Collins as she appeared in the TV drama *Dynasty* (Moore, 1981–1989), Lagerfeld once averred, did not have a direct effect on fashion, it being seen as too “hard” and aristocratic. However, mediated through pop singers such as Madonna and Sade, Collins’ stylized dresses garnered mass appeal (Pomazan, 1998, p. 23). Madonna’s and Sade’s self-presentation is descended from the Hollywood trope of the nightclub singer-immortalized by Rita Hayworth in the film *Gilda* (Vidor, 1946)-who makes every patron in the room feel integrated into her glamourous persona. As an example of how Lagerfeld followed this tradition of splicing together the upscale with the casual, consider his ready-to-wear “Pearl Trim Bell Sleeve Gown.” The garment’s design follows this casual-formal, light-heavy theme, being a simple, black polyester spandex evening dress fitted out with pearl-rimmed sleeves that flare out in a bell shape, affording its wearer the kind of staid glitz that befits a day at the market as much as a night at the opera.

### Wisdom

In the creative process, wisdom is the level at which products are sent out into the world, having been guided along the steps of their production by reflection on how consumers and collaborators can be co-opted into the values that these creations embody (Sternberg, 2003, pp. 178–179). Whereas creativity is more concerned with the flow of simple, bedrock processes that are judged and refined by intelligence, wisdom stands apart as the highest-order executive level wherein decisions are made concerning what to do, how to do it, and how well something was done (Sternberg, 1998). Though the three WICS levels overlap somewhat, wisdom can be distinguished by its dual concern both with the internal consistency of the values reflected in the creative product, and with the possible appeal these value choices may have with consumers.

Lagerfeld’s attainment of wisdom is evinced through his nightly vigil at his partner Jacques de Bascher’s bedside when the latter was in the last throws of AIDS-related illness in 1989. Though Lagerfeld had up to that point “hated sickness and death,” keeping “away from any colleague with a mild cold” (Bacqué, 2020, p. 191), when his close associate and quondam muse took ill, the famous designer overcame his aversion. Though he retained a frenetic pace of work even during this vigil period, Lagerfeld forced himself to sleep on a cot in a hospital room with a dying friend, forsaking the comforts of his lavish apartment. Though Lagerfeld’s actions certainly did not directly inspire his subsequent fashion productions, we can surmise that personal growth and value development were their unseen fruits.

Lagerfeld seems to have struggled with a potential drawback at the value-wisdom level, in that he often made offensive comments that were likely intended to generate interest in his creative, neo-decadent persona, but which often got him into hot water with journalists and other media figures who found his put-downs simply crude and mean-spirited. Examples abound, so it will suffice to mention just a few of Lagerfeld’s quips. Meryl Streep was called “cheap” (Van Der Meer, 2020); Adele was decried as “fat” (Fox, 2012). Even the deceased Princess Diana found herself at the business end of a Lagerfeld one-liner: “She was pretty, and she was sweet, but she was stupid” (Grigoriadis, 2006). However, in the case of Adele and a few others, Lagerfeld remained flexible enough to make apologies and other concessions (Misener, 2012; THR Staff, 2012). Overall, he remained firm in his fashion choices, and also in his self-fashioning as a Wildean fount of witticisms, though with an added understanding that offending the public’s sense of propriety in a deep and abiding manner is bad business.

### Conclusion and Recommendation

The aim of the study was to explore creativity in Karl Lagerfeld’s life and thereby reconstruct it by homing in on the theme of meaning-in-life. In this way, the study explores Lagerfeld’s core values by clarifying the link between his creative output and his attempt to foster meaning across his lifespan.

First, the interlinkages of creativity and meaning in life were discussed; secondly, the Big-C theory of Beghetto, Glăveanu and Runco and Robert Sternberg’s WICS model were called upon to forge a unique account of the life of Karl Lagerfeld in the context of creativity and meaning.

The study showed that Karl Lagerfeld’s creativity was evinced in his constant production of new ideas, his intelligent arrangement of his ideas, and his ability to sell his ideas to the broader public. Traces of mini-c, little-c, Pro-c and Big-C could be found across his lifespan, starting during his childhood with mini-c and little-c acts that blossomed into Pro-c and Big-C creativity during his years as a professional.

Through the study’s in-depth analysis of Lagerfeld’s life, it was established that he used, applied and developed creativity, intelligence and wisdom throughout his life. His facility for both witty anecdotes in interviews and unexpected twists in his fashion designs served to keep Lagerfeld’s name in the media, and also showed forth his uncanny ability to generate life-meaning in which the public was invited to participate. Although other associations were important, his relationship with his parents and particularly with his mother was paramount in creating a foundation for his later ability to create meaning through aesthetically- and entrepreneurially-informed decision-making.

Future research on specific phases of Lagerfeld’s life could bear much fruit, as they could make manifest the interconnections between specific events in the fashion designer’s life and the aesthetic productions coeval with them, the latter being explicated as solutions to developmental crises. Such studies should shed further light on Lagerfeld’s inner life by reading backwards from his fashion creations to the root of their inspiration in his thought-world. In general, future psychobiography should concern itself more with comparative analyses of creativity and meaning in extraordinary individuals from different socio-cultural backgrounds and professional contexts. Psychobiography in this comparative vein could foster new considerations of cross-cultural values and garner fresh insights into the nexus of individual creativity and social constraint.
